# The relationships of kinesiophobia and physical function and physical activity level in juvenile idiopathic arthritis

**DOI:** 10.1186/s12969-022-00734-2

**Published:** 2022-09-01

**Authors:** Leandra U. Woolnough, Logan Lentini, Sharareh Sharififar, Cong Chen, Heather K. Vincent

**Affiliations:** Department of Pediatrics and Physical Medicine and Rehabilitation, University of Florida, 1600 SW Archer Rd HD-409, PO Box 100296, Gainesville, FL 32610 USA

**Keywords:** Juvenile idiopathic arthritis, Physical function, Kinesiophobia, Pain interference, PROMIS

## Abstract

**Background:**

Kinesiophobia may hinder physical performance measures and functional quality of life in children with juvenile idiopathic arthritis (JIA). This study aims to quantify differences in physical function in patients with JIA compared to healthy controls, and determine the effects of kinesiophobia on physical function and physical activity.

**Methods:**

This was a comparative study of participants with JIA and healthy controls (JIA *n* = 26, control *n* = 17). All children with JIA had lower extremity joint involvement. Performance-based measures included gait speed, chair and stair navigation performance. Self-reported measures included Patient Reported Outcome Measurement Information System (PROMIS®) Physical Function Mobility, and Pain Interference and the Pediatric Functional Activity Brief Scale (Pedi-FABS). The Tampa Scale of Kinesiophobia (TSK-11) assessed patient fear of movement due to pain. Linear regression models were used to determine the contribution of TSK-11 scores on performance test and Pedi-FABS scores.

**Results:**

Gait speeds were 11–15% slower, chair rise repetitions were 28% fewer, and stair ascent and descent times were 26–31% slower in JIA than controls (*p* < .05). PROMIS® Physical Function Mobility scores were 10% lower and Pain Interference scores were 2.6 times higher in JIA than healthy controls (*p* = .003). TSK-11 scores were higher in JIA than controls (*p* < .0001). After controlling for covariates, TSK-11 scores explained 11.7–26.5% of the variance of regression models for stair climb time, chair rise performance and Pedi-FABS scores (*p* < .05).

**Conclusions:**

Children with JIA experience difficulty with tasks related to body transfers. Kinesiophobia is a significant contributor to the functional task performance and may impact clinical outcomes.

## Introduction

Juvenile Idiopathic Arthritis (JIA) is characterized by chronic joint pain, swelling, and limited range of motion in children. Our recent systematic review and other emerging evidence demonstrates that children with JIA adapt walking gait to produce more lower limb joint flexion, less hip and knee extension, and a more crouched guarded gait pattern even when children report low disease activity, pain and disability [1–3]. Furthermore, studies have shown the children receiving intraarticular corticosteroid injections to the foot retain motion deficits and gait aberrations months after the treatment, despite improvement in inflammatory symptoms [4–6]. Children with JIA have a decreased threshold to pain compared to healthy counterparts, which may contribute to central nervous system excitability and pain sensitization [5, 7–9]. Central sensitization can occur in JIA, and high levels of pain may contribute to negative psychological influence with pain experiences and maladaptation of physical movement and gait patterns to avoid pain [3, 10, 11].

Pain-related fear of movement, or kinesiophobia, plays a major role in the onset, persistence and exacerbation of chronic disability in a variety of musculoskeletal disorders [12]. Kinesiophobia can impact pain perception, proprioception, and functional performance across different conditions including frozen shoulder patellofemoral pain, temporomandibular disorders, and low back pain [13–16]. In patients with rheumatic conditions like arthritis, kinesiophobia predicts worse self-reported disability, physical functioning, efficacy for fall prevention, overall quality of life, and is related to worse objective scores for quadriceps muscle strength and knee flexion [17, 18]. Among children with chronic rheumatologic pain, however, the evidence is very limited with respect to the severity of kinesiophobia and relationships to daily activities of physical function, self-reported disability and quality of life. This is an important gap in the literature that needs to be addressed. As children with JIA mature into adults, it is likely that kinesiophobia is adversely shaping the physical movement patterns and subjective beliefs that they maintain throughout life. A clearer understanding of the relationships between pain, kinesiophobia and physical function performance will be critical in the development of new approaches to disease management and patient/family education.

The primary objective of this study was to determine the differences in kinesiophobia levels between children with JIA and healthy counterparts. The secondary objective was to quantify the relationships between objective and subjective functional task performance and specific gait metrics. We hypothesized that: 1) children with JIA would report higher levels of kinesiophobia than healthy controls; and 2) kinesiophobia scores would be inversely related to speed of functional task performance and gait speed, and to subjective scores of disability and impact on life.

## Methods

### Study design

This was a cross-sectional comparative observational study of children with JIA and age-matched healthy controls. All study procedures followed the requirements for the protection and treatment of human subjects as outlined in the Declaration of Helsinki. This study and all of its procedures were approved by the University of Florida Institutional Review Board (UFIRB) under the study number 201903394. All participants read and signed a UFIRB-approved informed consent document and children provided assent.

### Participants

Enrolled participants had to meet the following inclusion/ exclusion criteria: aged 7–21 years; free of pediatric diseases such as reactive arthritis or pigmented villonodular synovitis; no history of surgical instrumentation to joints under study (such as arthroscopy, synovectomy) or joint fusions and free of obesity (body mass index>95th percentile). Children with JIA were under the care of a board-certified pediatric rheumatologist. The diagnosis of JIA was made by International League of Associations for Rheumatology (ILAR) criteria with current or previous sacroiliac joint or lower extremity joint involvement [19]. JIA patients were recruited consecutively through the UF Pediatric Rheumatology clinic. Healthy controls were recruited through the UF Consent2Share program, which provides an easy way for patients and families to be notified about research studies for which they are qualified. Healthy control subject families were contacted via email.

### Clinical exam

Each patient underwent a physical exam which included assessment of each joint for swelling, range of motion and pain on range. Physician global assessment of disease activity (MD global) were also recoded. Laboratory studies that are part of standard care for JIA including antinuclear antibody (ANA) status, rheumatoid factor (RF), anti-citrullinated protein (anti-CCP) antibody, and HLA-B27 were recorded from the electronic medical record. The Childhood Health Assessment Questionnaire (CHAQ), is a widely-used, reliable and valid tool for the general physical functional assessment of children with JIA [20]. The CHAQ is comprised of eight domains (dressing, rising, eating, walking, hygiene, reach, grip, and activities), each containing different items that are scored from 0 to 3 points by level of difficulty (where 0 = “able to do with no difficulty” and 3 = “unable to do”). Global pain scores over the last 7 days were obtained using an 11-point numerical pain rating scale (NRS; 0–10 points) to estimate current pain burden [21].

### Experimental procedures

All participants completed a single testing session in the University of Florida Human Dynamics Laboratory in the Orthopedics and Sports Medicine Institute. Several self-reported and performance-based measures were obtained during this visit, including walking gait speed, functional tasks, patient-reported outcomes and kinesiophobia. Before testing height and weight were measured using a medical grade scale, and body mass index was calculated (BMI = body weight(kg)/height(m^2^)).

### Functional task performance and functional pain severity

Gait speed was captured on an instrumented treadmill (AMTI; Watertown MA) at self-selected and fastest tolerable speeds. Standardized instructions were provided to each participant for each test from written scripts. After acclimation for 3 min on the treadmill, the participant was asked to: 1) self-select a speed at which they felt represented a typical comfortable speed while taking a walk on the street, followed by 2) selection of the fastest comfortable speed they could achieve similar to ‘what they would use trying to speed up to catch a bus’, or ‘move in the hallway to avoid being late for a class.’

Two measures of leg power that were recommended for use by the OARSI initiative among people with arthritis were administered here: the 30-second chair rise test (repetitions in 30-seconds) and stair ascent-descent (time to completion) [22]. First, each child was instructed to rise from a chair (seat height 45 cm) with armrests and sit back down as many times as possible in 30 seconds. If the child’s feet were not able to touch the floor, a footstool was positioned under the foot so that in sitting position the knee was flexed at 90°. Children performed the tests with arms crossed in front of the body. The number of repetitions was recorded as the score. Second, the times to complete a climb up a standard flight of 12 stairs (each stair riser was 17.5 cm high) and to complete the descent back down were individually recorded by stopwatch. Children were instructed to climb stairs without the handrail. Slow times to complete the chair rise and stair climb and descent would be interpreted as impeded functional performance compared to faster times.

Pain elicited during the four functional task tests was described as functional pain [23]. Functional pain severity was obtained from each participant using the Wong-Baker FACES™ Pain Rating Scale, which is a visual pain scale ranging from 0 to 10 points. This scale was created for children to help facilitate communication and improve pain assessment [24].

### Patient reported survey outcomes

All participants completed paper-based surveys including Patient Reported Outcome Measurement Information System (PROMIS®; Pain Interference, Physical Function Mobility and Global Health instruments) and the Hospital for Special Surgery Pediatric Functional Activity Brief Scale (Pedi-FABS) [25]. PROMIS instruments assess physical, mental and social wellbeing in children and young adults across a variety of diseases [26]. We administered the pediatric short form versions for Pain Interference (Form 8a, v 2.0), Global Health (form 7, v 1.0), Physical Function and Mobility (Form 8a, v 2.0), and Upper Extremity (Form 8a v 2.0). The Pedi-FABS was administered to the participants and families to determine the child’s current participation over the past month in sports and physical therapy, sedentary behavior, frequency of performing different actions (e.g., running, cutting, endurance, jumping) and how different sports impact joint pain and swelling [25]. The Pedi-FABS is moderately related to PROMIS Physical Function and Mobility scores, and has excellent test-retest reliability (ICC = 0.91) and internal consistency (Cronbach’s alpha = 0.914) [25, 27]. PROMIS pain interference and Mobility scores are strongly related to CHAQ pain scores [28, 29].

**Fig. 1:**
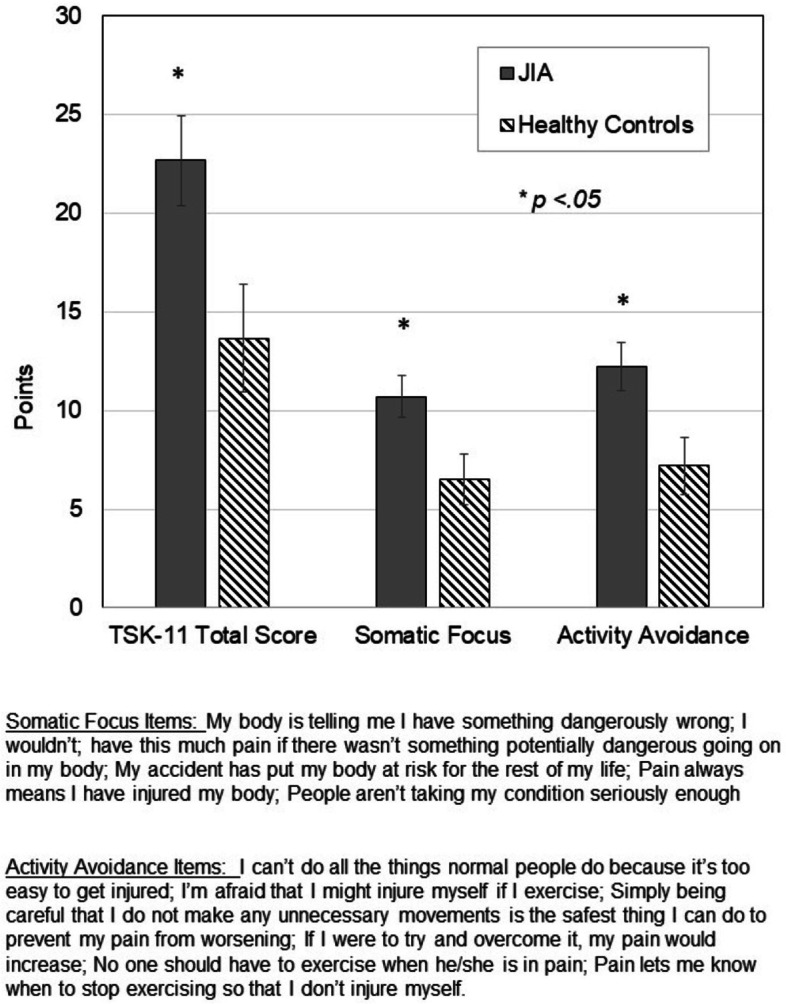
Tampa Scale of Kinesiophobia − 11 (TSK-11) scores and factor components, Activity Avoidance (TSK AA) and Somatic Focus (TSK SF). Values are means ± SD. * different than Healthy Controls at *p* < .05

The Tampa Scale of Kinesiophobia (TSK-11) is a brief, reliable, two-factor instrument comprised of 11 items that are scored on a 4-point Likert scale (ranging from 1 = ‘strongly disagree’ to 4 = ‘strongly agree) [30, 31]. Items are designed to assess fear of movement/ re-injury due to pain. Two factors, Somatic Focus (TSK11-SF) and Activity Avoidance (TSK-11 AA) were calculated from the sum of the scores from items in each factor (see Fig. 1 for description) [12]. Across a range of adult pain populations, the internal consistency of the two factors ranges from 0.64–0.80 [12].

### Statistics

Statistical analyses were conducted using SPSS version 26.0 (Chicago, IL; USA). For this relatively small sample, normality of data was visually inspected through distribution plots of each variable to ensure there were no obvious outliers. Descriptive statistics (means and standard deviations for continuous variables, frequencies and percentages for categorical variables) were calculated on all demographics, including race, sex, ethnicity, medication number and clinical characteristics for JIA. To determine whether group differences in patient reported measures, Welch’s t-tests were performed assuming unequal variance. To determine whether kinesiophobia predicted functional performance and physical activity, stepwise regression analyses were performed. Dependent variables were scores for 30-second chair rise, stair climb and descent, gait speed and Pedi-FABS. For each model sex and NRS Global pain level were entered into the models as covariates, and TSK-11 scores were entered last. The α level was set at .05 to establish statistical significance for all tests. Pearson correlations were performed between TSK-11 scores, performance-based test scores and Pedi-FABS scores; a Bonferroni correction was therefore applied for multiple comparisons for these four correlations with TSK scores; the α level was set as 0.012.

## Results

### Participant characteristics

Table 1 provides the characteristics of the children with JIA (*n* = 26) and healthy controls (*n* = 17). Overall, participants were well-matched for anthropometrics and demographics. Participants with JIA demonstrated various clinical features associated with the disease and elevated CHAQ scores. Among the children with arthritis, 6 were classified as oligoarticular arthritis, 3 were classified as extended oligoarticular arthritis, 4 were classified as psoriatic arthritis, 6 patients as seronegative polyarticular arthritis, and 7 were classified as enthesitis related arthritis. Disease duration varied between 0 and 15 years with a mean of 4 years. Global pain scores were 1.5 ± 1.8 points and 0.0 points in the JIA and healthy control groups, respectively. All children with JIA had lower extremity joint involvement, with the main site at the knee.

**Table 1:** Characteristics of children with JIA and healthy controls. Values are median and interquartile ranges (IQR) or percent of the group

	JIA (*n* = 26)	Healthy Control (*n* = 17)
Age (yrs)	14.5 (13–18)	15 (9–19)
Height (cm)	162.5 (141.25–172.5)	159.4 (150–170)
Body mass index (kg/m^2^)	22.7 (20.6–24.5)	20.7 (18.7–22.2)
Female (#, %)	15 (57.7)	10 (58.8)
Hispanic (#, %)	3 (11.5)	4 (25.0)
Active joint count (#)	1 (0–2)	–
cJADAS (points)	4.0 (0.5–9.0)	–
ANA positive (%)	52.7	–
HLA-B27 positive (%)	17.4	
RF negative (%)	95.6	–
Anti CCP Ab positive (%)	5.0	–
CHAQ	0.1875 (0–0.625)	0

*BMI* body mass index, *cJADAS* clinical juvenile arthritis disease activity score, *ANA* antinuclear antibody, *HLA* human leucocyte antigen, *RF* rheumatoid factor, *CCP Ab* citrullinated protein antibody, *CHAQ* Childhood Health Assessment Questionnaire

### Physical function, pain severity and patient-reported survey outcomes

Physical task performance scores, pain and patient reported disability and quality of life are presented in Table 2. Children with JIA demonstrated slower gait speeds (*p* < .05) in comparison with their healthy peers. Children with JIA performed 27% fewer chair rise repetitions than their healthy peers (*p* < .05). Similarly, stair climb and descent times were slower in children with arthritis (both *p* < .05). Children with arthritis described mild pain in all functional tasks including walking, rising from a chair, ascending and descending the stairs, but no participants had to halt or withdraw from the study activities secondary to pain.

**Table 2:** Functional task performance, functional pain and patient reported physical activity and disability. Values are means SD [95% CI]

Measure	JIA (*n* = 26)	Healthy Control (*n* = 17)	*p*
Gait speed, self-selected (m/s)	0.91 ± 0.18 [0.83–0.98]	1.07 ± 0.22 [0.95–1.20]	.015
Gait speed, fastest, comfortable (m/s)	1.41 ± 0.20 [1.31–1.51]	1.58 ± 0.17 [1.49–1.67]	.017
Pain with walking (points)	0.6 ± 1.2 [0.11–1.20]	0.0	
Chair rise repetitions (#)	12.5 ± 2.6 [11.4–13.5]	17.3 ± 3.2 [15.6–19.0]	<.001
Chair rise pain (points)	0.8 ± 1.3 [0.3–1.3]	0.0	
Stair climb time (s)	3.7 ± 0.8 [3.3–4.0]	2.9 ± 0.7 [2.6–3.2]	.003
Stair climb pain (points)	0.9 ± 1.5 [0.3–1.5]	0.0	
Stair descent time (s)	3.8 ± 1.0 [3.4–4.3]	3.0 ± 0.7 [2.6–3.4]	.011
Stair descent pain (points)	0.9 ± 1.4 [0.3–1.5]	0.0	
PROMIS Pain Interference (points)	12.5 ± 9.4 [8.5–16.6]	4.8 ± 4.0 [2.8–6.9]	.001
PROMIS Physical Function and Mobility (points)	31.8 ± 7.2 [28.9–34.8]	34.5 ± 4.1 [32.4–36.6]	.165
Pedi-FABS (points)	13.4 ± 9.3 [9.6–17.3]	16.8 ± 7.3 [13.1–20.6]	.238

Pain scores are from the 11-point Wong Baker Scale, where 0 = no pain and 10 = worst imaginable pain; *PROMIS* Patient-reported outcomes measurement information system, *Pedi-FABS* Pediatric Functional Activity Brief Scale

Children with arthritis self-reported significantly poorer physical function and worse pain interference by PROMIS survey measures compared to healthy children. Pain Interference scores were 2.6 times greater in participants with JIA than healthy comparators (*p* < .05). Physical function, mobility, and physical activity level, measured by the PROMIS Physical Function and Mobility and Pedi-FABS scores, were 8–20% lower in JIA, but did not achieve statistical significance.

### Tampa scale of kinesiophobia and relationship to physical function

**Fig. 2:**
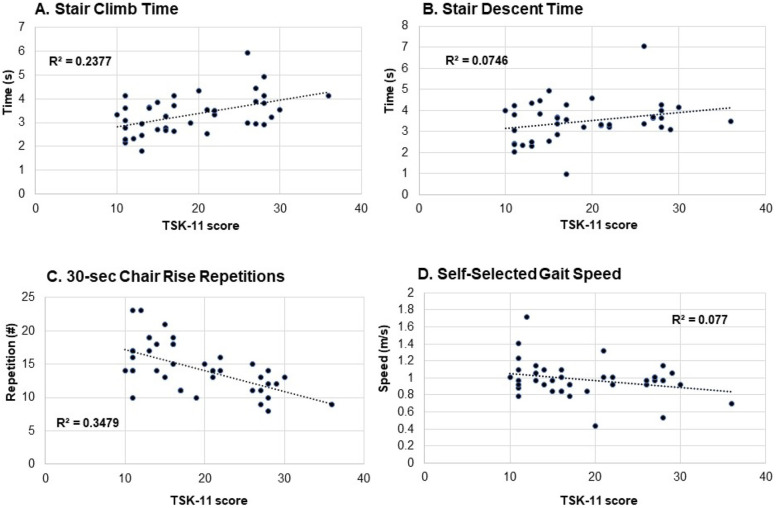
Scatter plots for TSK-11 total scores and **A** Stair climb time; **B** Stair descent time; **C** 30-second chair rise repetitions; and **D** Self-selected gait speed

TSK-11 scores and factor subscores are shown in Fig. 1. Children with JIA scored higher on overall TSK-11 scores and both TSK AA and TSK SF subscores than healthy children (all *p* < .05). Scatter plots of TSK-11 overall scores and each functional score (stair climb time, stair descent time, 30-second chair rise repetitions and gait speed) are provided in Fig. 2A-D. Significant correlations existed between TSK-11 and chair rise repetitions (r = −.590, *p* < .001) and stair climb time (*r* = .488, *p* < .002). Correlations did not achieve significance for stair descent time (*r* = .273; *p* = .088) and gait speed (*r* = −.276; p = .08). There were no significant associations between disease duration (*p* = 0.16) or measures of disease activity cJADAS (*p* = 0.98) and TSK-11 scores of kinesiophobia.

Results of the stepwise regression analyses are shown in Table 3. After controlling for sex and Global NRS pain level, TSK-11 scores were found to be significant contributors to chair rise performance (explained 26.5% of the variance of the model; *p* < .001), stair climb time (explained 11.7% of the variance of the model; *p* = .015) and Pedi-FABS scores of physical activity level (explained 25.6% of the variance of the model; *p* < .001).

**Table 3:** Stepwise regression analyses for functional task performance and physical activity level

	R	R^2^	R^2^ change	*p*-value	B (CI)
**Model 1. Self selected gait speed**					
Sex	.172	.030	.030	.282	.073 (−.062–.208)
Pain	.365	.134	.104	.039	−.042 (−.081 - -.002)
TSK-11	.400	.160	.026	.291	−.005 (−.015–.005)
**Model 2. Chair Rise Performance (repetitions in 30 seconds)**					
Sex	.229	.053	.053	.144	1.694 (−.606–3.995)
Pain	.328	.107	.055	.130	−.537 (−1.240–.165)
TSK-11	.611	.373	.265	<.001	−.295 (−.445 - -.146)
**Model 3. Stair Climb performance (time to climb one flight)**					
Sex	.185	.034	.034	.241	−.297 (−.800–.207)
Pain	.444	.197	.163	.008	.201 (.056–.345)
TSK-11	.561	.314	.117	.015	.043 (.009–.076)
**Model 4. Stair Descent performance (time to descend one flight)**					
Sex	.102	.010	.010	.525	.202 (−.435–.839)
Pain	.371	.138	.127	.023	.218 (.031–.404)
TSK-11	.379	.144	.006	.605	.012 (−.035–.060)
**Model 5. Physical Activity levels (Pedi FABS score)**					
Sex	.083	.007	.007	.600	−1.431 (−6.905–4.044)
Pain	.194	.038	.031	.272	−.934 (−2.630–.761)
TSK-11	.542	.294	.256	<.001	−.675 (−1.043–.307)

Dependent variables in each model are shown. Sex and global NRS pain scores were added to each model first, followed by Tampa Scale of Kinesiophobia Scale (TSK-11 score) last. *B* unstandardized coefficient, *CI* confidence interval

## Discussion

The purposes of this study were to determine the differences in kinesiophobia levels between children with JIA and healthy counterparts, and to determine the relationships self-reported and performance-based tasks and gait metrics. Our hypothesis was supported that children with JIA have higher levels of kinesiophobia and pain interference than healthy controls. Moreover, the TSK-11 score was a significant contributor to the repeated chair rise and stair climb performance scores and to physical activity level. In contrast to our hypotheses, the TSK-11 score was not significant contributor to the variance of regression models for gait speed or stair descent time. These findings could suggest that specific types of function and engagement in physical activity are differentially impacted by kinesiophobia in children with JIA.

Patients with active or previous disease involvement of the sacroiliac joint and/or the joints of the lower extremities were enrolled in this study. The subtypes of arthritis were distributed between oligoarticular (persistent and extended), psoriatic, enthesitis related arthritis and seronegative polyarticular arthritis subtypes. In this cohort, the disease duration and active joint count varied greatly between 0 and 15 years and 0 and 6 active joints, respectively, giving a broad spectrum of disease. In terms of serological markers, the majority of these patients had a positive ANA and a negative rheumatoid factor (Table 1). Despite the variations in disease activity levels, PROMIS Pain Interference sores were 2.6 times higher among children with JIA (Table 2). This perceived interference corresponded to slower gait speeds and limitations in performance-based tests compared to their peers. A slower, careful or crouched gait has been demonstrated in children with JIA with varying states of disease [2, 3, 32]. In our present study, children with arthritis required longer times for stair navigation and could not achieve as many chair rises as healthy children (Table 2). Stair navigation is a validated measure of function and functional limitation in adults with osteoarthritis [33, 34], and has shown value here in children with arthritis and being sensitive to the presence of disease. Stair navigation, particularly descent, produces large forces and moments in the joints of the lower extremities [35] which could evoke pain during movement.

Perceptions of pain interference, coupled with kinesiophobia likely contribute to functional performance or physical activity limitations depending on the task. The concept of fear of movement has scarcely been evaluated in children [36] and is a novel field of exploration for children with JIA. The TSK-11 was most strongly related to movements involving vertical transfer of body weight and to participation in stressful movement patterns (Pedi-FABS scores incorporating running, cutting, pivoting, decelerating; Table 3). In this pediatric population, it is not clear whether avoidance of activity due to fear reduces functionality or engagement in exercise, structured sport or competitive activities. Among adults with osteoarthritis, lower knee flexion/ extension strength is associated with lower physical activity level, and this relationship is mediated by kinesiophobia [37]. Moreover, kinesiophobia constructs appear to reflect patient perception that painful activity will produce damage to the body and will increase suffering and/or functional loss among adults [38]. In children, how Somatic Focus and Activity Avoidance constructs represent their perceptions of pain and movement are not yet understood. Given the fluctuating nature of JIA disease activity, pain symptoms functional tolerance, a child’s interpretation and processing of pain over time may be very different than an adult with chronic pain or acute injury. Prospective tracking of kinesiophobia throughout childhood, in parallel with functional testing and physical activity tracking, would provide a better understanding of the role of fear of movement on life activity. Another methodological consideration is to account for BMI during childhood in our measurement battery, to contextualize performance and kinesiophobia responses over time.

The literature on physical activity levels in JIA is mixed. Our findings are similar to accelerometry studies that show physical activity levels and participation in patients with oligoarticular and polyarticular JIA are comparable to their healthy peers [39], but are in direct contrast with other studies reflecting decreased physical activity levels in children with arthritis despite adequate disease control [11, 40, 41]. Of note, we found wide variation in Pedi-FABS scores indicating that some children with JIA are very active despite symptoms and disease duration, whereas others are sedentary (Table 2). Our regression results (Table 3) indicate that higher activity levels are related to lower kinesiophobia. It is not clear whether: 1) active children become less fearful over time with more exercise exposure, 2) active children with JIA would have always been active irrespective of JIA because of the positive experience they receive from exercise, or 3) kinesiophobia drives children to avoid activity. We surmise that all three patterns may occur in this population depending on the child. Most studies do agree that children with JIA are not as engaged in moderate-to-vigorous physical activity as their healthy counterparts which is not completely explained by pain or objective measures of inflammation [39, 40]. The important point is to identify children experiencing debility and physical activity avoidance and intervene to address kinesiophobia. From the developmental perspective, understanding and addressing fear of movement and encouraging involvement in different types of exercise can improve strength, cardiovascular capacity and psychosocial health [42, 43]. Engagement in physical activity is a complex challenge in this population, as physical activity level in JIA has not been found to be related to functional ability, disease duration and disease activity [44]. Additional studies are necessary to determine from a wider view how kinesiophobia may be driving treatment efficacy and participation in physical activity.

### Limitations and strengths of this study

There are a few limitations of the current study. First, the concept of fear of movement is unlikely to be stagnant throughout childhood arthritis, and may evolve with growth, maturity and disease activity. A longitudinal, population-based Nordic study found that school absences and participation in physical education was the greatest early in the disease course, higher levels of disease activity, and among children with enthesitis-related arthritis subtype [45]. We present here a snapshot in time of the patient experience and a relatively small patient group. Contributions of kinesiophobia to physical activity and physical function would be better clarified with longitudinal evaluation at multiple time points of disease activity. Second, there is currently no ‘gold standard’ for assessing changes to fear of movement [30], particularly among children. There are few measures that address specific constructs of fear of participation in children. Unlike the PROMIS and Pedi-FABS which were designed for the pediatric population, the TSK-11 is a validated measure of kinesiophobia in the adult population that has not yet been validated in children. The language in the TSK-11 would benefit from language simplification and adaptation tailored to the pediatric population. The Pediatric PROMIS versions were used for all participants even up to 21 years, and future work may test whether adult PROMIS versions may be more responsive to JIA.

Additional limitations include potential selection bias. For example, sampling bias excluded children who had upper limb involvement but not lower limb, which decreases the generalizability of these functional tests for all children with JIA. Volunteer bias children who agreed to participate may be more motivated and excited to engage the testing and may perform physically and psychologically better than children who are not motivated. Additionally, the age range included in this preliminary study included ages 7–21 years which includes both school aged children and young adults. In future iterations of this work, we will narrow our age range to cap at 16–18 years to control for potential impacts of growth and development on physical function. This cohort included children with body mass indexes of 15–31 kg/m^2^, which may introduce potential impacts of obesity on gait mechanics and physical function.

Strengths of this study include rigorous measures of physical function, validated and reliable testing measures, a well-matched healthy control group and a population that represents the general JIA population.

## Conclusions

Children with JIA generally demonstrate lower self-reported and performance-based scores in areas of mobility and body weight transfer. In this cohort, children with JIA demonstrated kinesiophobia, which contributed directly to performance of body weight transfer motions and to physical activity levels. Kinesiophobia may inform clinical care through the reflection of the patient experience of living with JIA.

## Data Availability

The data that support the findings of this study are available on request from the corresponding author.

## Abbreviations

ANA: Antinuclear antibody
CHAQ: Childhood Health Assessment Questionnaire
JIA: Juvenile Idiopathic Arthritis
NRS: Numerical Rating Scale
PROMIS: Patient Reported Outcome Measurement Information System
Pedi-FABS: Pediatric Functional Activity Brief Scale
TSK-11: Tampa Scale of Kinesiophobia
TSK AA: Tampa Scale of Kinesiophobia Activity Avoidance
TSK SF: Tampa Scale of Kinesiophobia Somatic Focus
